# Task Shifting for Non-Communicable Disease Management in Low and Middle Income Countries – A Systematic Review

**DOI:** 10.1371/journal.pone.0103754

**Published:** 2014-08-14

**Authors:** Rohina Joshi, Mohammed Alim, Andre Pascal Kengne, Stephen Jan, Pallab K. Maulik, David Peiris, Anushka A. Patel

**Affiliations:** 1 The George Institute for Global Health, Sydney, Australia; 2 University of Sydney, Sydney, Australia; 3 The George Institute for Global Health, Hyderabad, India; 4 Medical Research Council, Cape Town, South Africa; 5 University of Oxford, Oxford, United Kingdom; University of Massachusetts Medical School, United States of America

## Abstract

**Background:**

One potential solution to limited healthcare access in low and middle income countries (LMIC) is task-shifting- the training of non-physician healthcare workers (NPHWs) to perform tasks traditionally undertaken by physicians. The aim of this paper is to conduct a systematic review of studies involving task-shifting for the management of non-communicable disease (NCD) in LMIC.

**Methods:**

A search strategy with the following terms “task-shifting”, “non-physician healthcare workers”, “community healthcare worker”, “hypertension”, “diabetes”, “cardiovascular disease”, “mental health”, “depression”, “chronic obstructive pulmonary disease”, “respiratory disease”, “cancer” was conducted using Medline via Pubmed and the Cochrane library. Two reviewers independently reviewed the databases and extracted the data.

**Findings:**

Our search generated 7176 articles of which 22 were included in the review. Seven studies were randomised controlled trials and 15 were observational studies. Tasks performed by NPHWs included screening for NCDs and providing primary health care. The majority of studies showed improved health outcomes when compared with usual healthcare, including reductions in blood pressure, increased uptake of medications and lower depression scores. Factors such as training of NPHWs, provision of algorithms and protocols for screening, treatment and drug titration were the main enablers of the task-shifting intervention. The main barriers identified were restrictions on prescribing medications and availability of medicines. Only two studies described cost-effective analyses, both of which demonstrated that task-shifting was cost-effective.

**Conclusions:**

Task-shifting from physicians to NPHWs, if accompanied by health system re-structuring is a potentially effective and affordable strategy for improving access to healthcare for NCDs. Since the majority of study designs reviewed were of inadequate quality, future research methods should include robust evaluations of such strategies.

## Introduction

Non communicable diseases (NCDs) disproportionately affect low and middle income countries (LMIC) where nearly 80% of NCD related deaths occur [1]. Furthermore, NCD in LMIC occurs at a much younger age, thereby contributing to loss of potential years of healthy life, as well as impacting on economic productivity. Over the coming decades, the prevalence of NCD is expected to increase as the population ages [2]. In 2028, it is anticipated that global cost of Cardiovascular Disease (CVD) will be US$47 trillion; this includes direct healthcare costs and productivity loss from disability or premature death, and time loss from work because of illness or the need to seek care [3].

In most countries, primary healthcare physicians are the first point of contact and the main providers of healthcare for individuals with NCDs. In LMICs too few doctors exist and physician workforce disparities for rural and remote regions are very substantial [4], [5], [6] In low income countries – those with an annual gross national income per capita below $1,035, 0.3 physicians are available for every 1000 population, compared to 1.2 physicians for every 1000 population in MIC, and 2.0 in upper middle income countries.

In this context, there is a need to develop an alternative workforce that is structured around the community and consumer needs. Task-shifting describes a situation where a task normally performed by a physician is transferred to a health professional with a different or lower level of education and training, or to a person specifically trained to perform a limited task only, without having formal health education [7]. Task-shifting may be facilitated by medical technology, which standardizes the performance and interpretation of certain tasks, therefore allowing them to be performed by non-physicians or technical assistants instead of physicians. This has typically been done in close collaboration with the medical profession [8]. Task-shifting can potentially result in cost and physician time savings without compromising the quality of care or health outcomes for patients [9], [10], [11], [12]. A study from Uganda reporting the potential impact of task-shifting on the costs of antiretroviral therapy and physician supply found that the estimated annual mean costs of follow-up per patient were US$31.68 for physician follow-up, US$24.58 for nurse follow-up and US$10.50 for pharmacist follow-up [13]. It is also potentially an efficient way of reorganising the workforce by ensuring better specialisation of tasks, allowing physicians to focus on the jobs that cannot be otherwise delegated. A study from Rwanda showed that task-shifting from a physician-centred to a nurse-centred model for antiretroviral therapy reduced the demand on physician time by 76% [9].

Task-shifting in healthcare dates back to the 1970s–80s where auxiliary nurses in The Democratic Republic of Congo took on the role of providing healthcare due to a shortage of physicians. This allowed the few physicians available to use their time and expertise to manage people with more complicated disease. Other LMICs in Africa and South Asia have trialled this approach for childhood conditions [14], [15] and infectious diseases [16]. A Cochrane review assessing the performance of NPHWs on maternal and child health in indicated that task-shifting had a benefit in promoting immunisation, breastfeeding, improving tuberculosis outcomes and reducing childhood morbidity and mortality when compared to usual care [17]. There is now growing evidence from countries in Africa that have experienced task-shifting for anti-retroviral therapy to curb the HIV-AIDS epidemic. A systematic review of task-shifting for HIV care in Africa showed that task-shifting offered cost effective and high quality care to more patients than a physician-centred model [18]. There are very few studies that have focussed on the role of NPHWs in managing NCDs in LMICs, with most of these studies focussing on a single risk factor or disease [7], [19] rather than on integrated disease management. We sought to systematically appraise the literature to assess the effectiveness, cost-effectiveness and barriers to task-shifting for the management and prevention of NCDs in LMICs.

## Methods

We conducted a systematic review of studies that involved task- shifting of the management of NCDs to NPHW in LMIC. The search was conducted from 26^th^ May to 13^th^ June 2013. For the purpose of this review, a NPHW was defined as a lay healthcare worker with no formal medical training or nurses. The term NCDs defined a range of chronic non-infectious conditions including cardiovascular disease, diabetes mellitus, hypertension, cancer, chronic obstructive pulmonary disease, neurological conditions and mental health. A search strategy with the following terms was used “Task-shifting”, “Non-physician healthcare workers”, “Community healthcare worker”, “Hypertension”, “Diabetes”, “Cardiovascular disease”, “mental health”, “depression”, “chronic obstructive pulmonary disease”, “respiratory disease”, “cancer”. The following databases were reviewed: Medline via PubMed and the Cochrane library. Two authors (RJ and MA) reviewed the literature and extracted the data independently. If there was a disagreement about the inclusion of a paper, a third author (PKM) adjudicated. The references of all the included papers were checked for additional relevant papers. If a study was reported in two journals, the article with the maximum detail was chosen. In some cases, details were gathered from more than one article. Table 1 highlights the inclusion and exclusion criteria of the systematic review. The review was limited to peer reviewed, community-based studies conducted in LMICs and studies that involved an intervention. Studies involving health education or health promotion and hospital-based studies were excluded. Only English language reports were considered.

**Table 1:** Inclusion and Exclusion Criteria.

Inclusion criteria	Exclusion criteria
Studies where a task usually performed by physicians is shifted to a different cadre of health care provider	Studies involving health education or health promotions
Disease conditions limited to non-communicable disease and mental health cardiovascular disease, diabetes mellitus, hypertension, cancer, chronic obstructive pulmonary disease, respiratory mental health.	Hospital based studies
Studies conducted in low and middle income countries	
Intervention studies – RCTs/before after studies and quasi-experimental studies	
Community based studies	
Peer reviewed articles	
Articles in English Language	

The quality of studies was assessed on criteria such as design of the study, method of randomisation and sources of bias. No study was excluded based on the quality of the paper. A meta-analysis was not done due to high levels of heterogeneity between studies on the task-shifting model under evaluation, types of patients and outcomes used.

## Results

### Characteristics of studies

**Figure 1:**
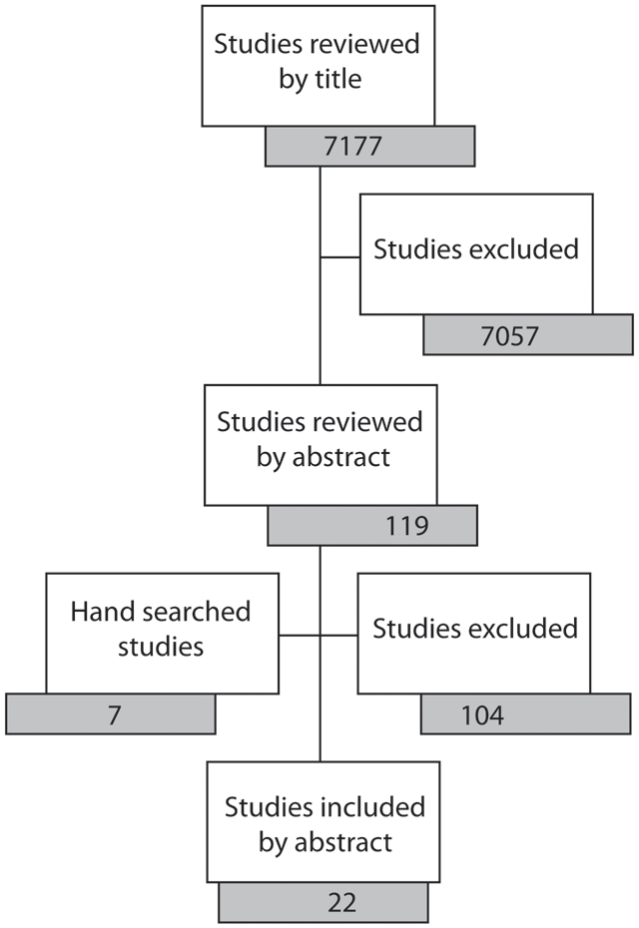
The process of identifying relevant papers.

Our search generated 7176 articles of which 22 were included in the review (Figure 1). Six studies were conducted in Cameroon, six in India, two in South Africa and one each in China, Ethiopia, Kenya, Pakistan, Philippines, Tanzania and Zimbabwe. Nine studies were based in rural regions, six in urban and seven included both rural and urban regions. Seven studies involved task-shifting for the management of hypertension and cardiovascular diseases, five for diabetes, six for mental health, four for neurological conditions, two each for the screening and management of respiratory diseases and five for the screening of cancers. Tasks were shifted from physicians to midwives [20], [21], nurses [19], [21], [22], [23], [24], [25], [26], [27], [28], [29], or health workers [30], [31], [32], [33], [34], [35]. The comparator for randomised control trials (RCTs) was usual healthcare in 5 studies [31], [33], [34], [35], [36], and health education in 2 studies [21], [32]. Table 2 illustrates the characteristics of the studies included in this review and the tasks performed by the NPHWs in each of the studies.

**Table 2:** Studies included in the review.

Author, year	Country	Diseaseaddressed	Studytype	Intervention	Outcome	Issues	Cost effectiveness analysis
**Task shifting for screening of disease**							
Warnakulasuriya KAAS, 1984[20]	Sri Lanka	Oral cancer	Before-after	NPHWs trained in screening of oral cancer. Comparator: Usual care	NPHWs could screen individuals with oral lesions and refer them to specialists	50% lost to follow up	Yes
Gajalakshmi CK, 1996[52]	India	Cervix cancer	Before-after	Trained NPHWs conducted visual inspection of cervix for cancer screening Comparator: Usual care	NPHWs could reliably screen women for cervix cancer	None reported	No
Feksi AT, 1991[30]	Kenya (rural)	Epilepsy	Before-after	Patients screened and monitored by NPHWs. Treatment initiated by a psychiatrist. Comparator: Usual care	Decreased episodes of seizures among participants on drugs. 53% seizure free in 6–12 month follow up	None reported	No
Sankaranarayanan R, 2005[31], [39], [41]	India	Oral cancer	RCT*	NPHW led screening of oral cancer. Comparator: Usual care	NPHWs could screen individuals with oral lesions and refer them to specialists	None reported	Yes
Pisani P (2006) [21]	Philippines	Breast cancer	RCT	NPHWs examined women for breast lumps and masses and referred them to hospital diagnostic centres. Comparator: Usual care	2.5% screened positive.	Of those who detected positive for a lump, 42.4% refused follow up and 22.5% could not be traced	No
Dinshaw K (2007) [32]	India	Cancer (breast and cervix)	RCT	NPHWs examined women for breast lumps and did visual inspection of cervix with acetic acid. Follow up and referral was provided Comparator: Health education	Average compliance of 73% was achieved when women were referred for a breast mass and 78.9% when they were referred for cervix cancer.	None reported	No
Kar SS, 2008[6]	India (rural, urban and slum)	Cardiovascular disease	Before-after	NPHWs trained in WHO protocol for CVD risk assessment. Comparator: Usual care	Increase in knowledge of NPHWs regarding CVD risk factors and symptoms. Increase in referral of individuals with raised SPB. Decrease in SBP during follow up. Significantly higher reports of intention to quit tobacco (60.3% vs 25.5%) and regular intake of anti-hypertensive medication (58.3% vs 34.8%)	None reported	No
Adams JL, 2012[29]	Tanzania	Depression (in the context of HIV/AIDS)	Before-after	NPHWs screened individuals and the medical officer initiated treatment. Titration of dose was done by the medical officer. Comparator: Usual care	Depression score (PHQ-9) decreased over the course of the 12 week intervention from 19.8 to 8.1	Drug supply stopped between week 8 and 10	No
Joshi R, 2012[35]	India (rural)	Cardiovascular disease (CHD and Stroke)	cRCT#	NPHWs trained to opportunistically screen individuals at high risk of developing CVD. Algorithm based care Comparator: Usual care	The proportion of high risk individuals screened was 12% more in intervention villages. Agreement between the recommendations made by the trained NPHW and physicians was 88.5%.	None reported	No
**Task shifting for screening and/management of disease**							
Coleman R, 1998[22]	South Africa (rural & urban)	Hypertension and diabetes (also epilepsy and asthma)	Before-after	Protocol developed based on World Health Organisation guidelines. Patients initially screened by a doctor, and followed up by NPHWs. Comparator: Usual care	BP controlled in 68% of patients, blood glucose controlled in 82% of patients with type 2 diabetes mellitus, Better adherence	High attrition of patients	No
Berhanu S, 2002[23]	Ethiopia	Epilepsy	Before-after	Training of NPHWs for screening and management of epilepsy. Treatment was initiated by physicians. Comparator: Usual care	Improved identification and treatment of individuals with epilepsy	None reported	No
**Task shifting for management of diseases**							
Rahman A (2008) [33]	Pakistan	Mental health (depression)	RCT	Psychological intervention by NPHWs. Comparator: Health education	Significant reduction in depression score	None reported	No
Chibanda D, 2011[37]	Zimbabwe	Mental health (depression)	Before-after	NPHWs screen individuals for common mental disorders and gave 6 sessions of problem solving therapy. Comparator: Usual care	Mean depression score (Shona Symptom Score) fell from 11.3 to 6.5 after 3–6 sessions	None reported	No
Patel V, 2011[10], [34]	India	Mental health	cRCT	NPHWs screened individuals for common mental disorders and offered 6 sessions of therapy. Patients referred to the GP or a psychiatrist, if required. Comparator: Usual care	In patients attending public facilities, 24% reduction in depression, 34% reduction in screen positive group and 57% reduction in the sub-threshold group	None reported	Yes
Kaufman JA, 2012[28]	China (rural)	Mental health (depression)	Before-after	NPHWs counselled participants, Comparator: Usual care	Improvement in anxiety score (CARED score) from 29.8 at baseline to 23.8 at the end of first follow-up	None reported	No
Petersen I, 2012[38]	South Africa (rural)	Mental health (depression)	Before-after	Group based Interpersonal therapy by NPHWs under supervision of a mental health counsellor Comparator: Usual care	Significant reduction in depressive symptoms. Lower BDI score (depression scale) in intervention group compared to the control group	None reported	No
**Task shifting for management of diseases including prescription**							
Kengne AP, 2008[24]	Cameroon (rural and urban)	Epilepsy	Before-after	Protocol driven treatment of patients. NPHWs allowed to prescribe. Comparator: Usual care	Significant decrease in the number of days/month with seizures	None reported	No
Kengne AP, 2008[25]	Cameroon (rural)	Asthma	Before-after	Training of NPHWs for diagnosis and management of asthma. Monthly visit by physician. Patients screened and managed by nurses. Comparator: Usual care	Increase in number of days without asthma attack	41% lost to follow-up	No
Kengne AP, 2009[26]	Cameroon (rural & urban)	Hypertension and diabetes	Before-after	Training of NPHWs for diagnosis and management of hypertension and diabetes. Clinical management algorithm Comparator: Usual care	BP decreased by 5.9/3.3 mmHg. Fasting glucose decreased by 1.6 mmol/l	High attrition of patients	No
Kengne AP, 2009[27]	Cameroon (rural & urban)	Hypertension	Before-after	Training of NPHWs for diagnosis and management of hypertension. Clinical management algorithm Comparator: Usual care	BP decreased by 11.7/7.8 mmHg.	High attrition of patients	No
Labhardt ND, 2010[19]	Cameroon (rural)	Hypertension and diabetes	Before-after	Training of NPHWs Provision of equipment (sphygmomanometer, stethoscopes, blood glucose meters) Management of hypertension with drugs Comparator: Usual care	100% retained equipment; 70% had functional blood glucose meter; 96% antihypertensives, 72% oral anti-diabetics. Knowledge of NPHWs significantly improved. BP decreased by 22.8/12.4 mmHg and blood sugar by 3.4 mmol/l	Changes in staff, Low case detection, High attrition of patients	No
Labhardt ND, 2011[36]	Cameroon (rural)	Hypertension and diabetes	RCT	NPHW led care. Group 1. Treatment contract between patient and nurse + free medication for a month for every 4 months of consecutively attended follow up visits, Group 2. Treatment contract + letters reminding patients for a visit. Comparator: Usual care	Retention rates 60% and 65% in groups 1 and 2, 29% in control group;	50% lost to follow up across the 3 arms	No

*Randomised Control Trial # Cluster Randomised Control Trial.

### Quality of studies

Seven of the 22 studies were randomised controlled trials, and the remaining were before-after studies. The sample size varied from 21 [29] to 151,538 [32]. Eight of the 22 studies did not discuss sources of bias or limitations of the study findings and did not use appropriate statistical analysis. Some studies reported challenges faced during the study where more than 40% of the patients were lost to follow-up [19], [20], [25] and one study had to be terminated prematurely due to poor participation [21].

### Does task-shifting improve health care effectiveness?

#### Process outcomes

The studies reviewed suggested that trained NPHWs can successfully screen individuals in the community for various NCDs such as asthma [22], cancer [20], [21], [31], [32], cardiovascular disease [35], hypertension [19], [22], [27], [36], diabetes [19], [22], [26], [36], depression [28], [29], [33], [34], [37] and epilepsy [22], [23], [30]. Studies which permitted NPHWs to prescribe drugs showed that trained NPHWs can effectively treat patients according to study protocols for conditions such as asthma [22], [25], hypertension [19], [22], [27], [36], diabetes [19], [22], [26], [36], depression [29], [33], [34], [37], [38] and epilepsy [24], [30]. Out of the 11 countries that were included in this study, 6 countries from sub-Saharan Africa had legislation giving authority to nurses to prescribe from a restricted list of drugs. Several studies reported improved access to healthcare at the community level, although the metric to evaluate access was often not described [19], [22], [26], [30].

#### Disease outcomes

A before-after study from rural South Africa showed that trained NPHWs, with the help of treatment protocols and working without the input of physicians, could achieve control of 68% of patients with hypertension, 82% with diabetes and 84% with asthma [22]. Four studies which focussed on depression related interventions all reported significant reduction in depression scores [29], [34], [37], [38] and improvement in anxiety [28]. The three studies that focussed on epilepsy showed that NPHWs could screen individuals with epilepsy [23], [24], [30] and treat patients with the guidance of physicians [24], [30]. These studies reported a significant increase in the seizure free days and decrease in the number of episodes among patients on medications [23], [24], [30].

#### Treatment concordance

Five studies specifically examined concordance between physicians and NPHWs for diagnosis [35], screening [20], [21], [32], [39], or management,[35] of NCDs. Overall all of these studies demonstrated high level of agreement between NPHWs and physicians. Two cancer screening studies from India and Sri Lanka showed 89% agreement in the clinical diagnosis made by NPHWs and physicians [20], [40]. A study from rural India reported that the recommendations for drug therapy made by NPHWs, guided by algorithms were the same as those made by physicians in more than 87% in cases of suspected stroke and myocardial infarction [35].

### Does task-shifting improve cost-effectiveness?

Only two studies reported cost-effectiveness outcomes [10], [41]. A study in India that evaluated task-shifting to screen and refer individuals with precancerous and cancerous oral lesions, reported it to be cost-effective, costing under US$6 per person eligible for screening over the course of 9 years [41]. The second study involved a task-shifting intervention for the screening and treatment of anxiety and depressive disorders in primary care settings in India. The time costs for the patients and families and the total cost were lower and the health outcomes were significantly higher for the intervention arm of the study compared to the control arm. Cost-effectiveness analysis indicated that the use of NPHWs in the care of patients with mental health disorders in the public primary health care facilities was cost-saving [10].

### What are the barriers and enablers that influence the effectiveness of task-shifting initiatives?

#### Enablers for task-shifting

Health system factors such as training of NPHWs, provision of algorithms [35], protocols and guidelines for screening, treatment and drug titration [22], and availability of drugs [22] were factors that were determined to aid in the success of the task-shifting intervention. Several studies had a training component specifically designed for NPHWs which involved development of algorithms and protocols and training for screening, diagnosis, management and follow up for various diseases [19], [21], [23], [26], [27], [32], [33], [34], [35], [38], [39]. Two studies reported significant changes in the knowledge level of NPHWs as a result of training and supervision [6], [19]. A study from Cameroon showed that the knowledge regarding the choice of correct antihypertensive drugs improved substantially after training (from 17% to 94%) and remained high 2 years after the intervention (95%) [19], while another study from India indicated that the knowledge levels of NPHWs for CVD increased from 47% to 93% after a 4-day training program [6]. The provision of diagnostic and management protocols with treatment algorithms was another key element that determined the success of some task-shifting studies [35], [42]. Two studies from South Africa and Cameroon developed detailed protocols for hypertension, diabetes and asthma management based on WHO and international guidelines [22], [42], similar protocols were developed for cancer screening in The Philippines and India [21], [31], [32], and CVD screening and management in India [6], [35]. Several studies had a task-sharing model where physicians were available for complicated cases [24], for confirming the diagnosis and initiating treatment for diseases such a breast and cervical cancer [32] and cardiovascular disease [35], and monitoring the management for conditions such as epilepsy [23], [25]. A c-RCT in rural Cameroon showed that nurse-led facilities could retain patients at the end of 1 year with free drugs and reminder letters. The retention rates in the two intervention arms were 60% and 65%, respectively, compared with 29% in the control group [36].

#### Barriers to task-shifting

Some of the barriers to successful task-shifting highlighted in these studies included problems with staff retention, irregular drug supply, and unavailability of equipment. A study in Cameroon reported challenges with staff retention with only 48% of the trained NPHWs retained at the end of the 2 year study [19]. Some primary health centres did not have equipment to measure blood pressure or blood glucose, and did not have protocols or guidelines for management of NCDs [19], [22], [35]. Availability of drugs was another challenge faced by some studies. One study in Tanzania had to interrupt treatment of patients for 2 weeks due to failure of drug supply [29], while two others had to directly provide drugs for the patients as the primary health care centre did not store sufficient drugs for the management of NCDs [19], [22]. A cluster randomised control study in rural India that showed NPHWs could screen individuals at high risk of CVD with the help of an algorithm, failed to demonstrate any effects on outcomes such as number of drugs prescribed or parameters such as blood pressure and lipid levels. In order to obtain treatment, patients had to visit a physician as NPHWs did not have authority to prescribe medications [35].

## Discussion and Conclusion

The acute shortage and mal-distribution of the health workforce is a major obstacle in achieving better health outcomes for the prevention and control of NCDs in LMIC. Historically, re-organising the healthcare workforce for the delivery of maternal and child health made significant improvements in the outcomes [43]. More recently, task-shifting has proved to be a viable and cost-effective option for the management of HIV-AIDS in Sub-Saharan Africa [12]. High income countries like the UK, USA and Australia have somewhat re-engineered their workforce for better efficiency of health care. For example, tasks such as taking blood samples, which were performed by physicians several decades ago, have been shifted to NPHWs like phlebotomists who specialise in taking blood samples, thereby freeing up physician time to do other important tasks involved in patient management. Nurse practitioners in these countries are increasingly adopting many aspects of healthcare delivery that were traditionally the domain of physicians.

Task-shifting alone will not solve the problem of NCD control in LMICs. Re-engineering the health workforce will need to be implemented along with changes in the health system including training of NPHWs in these new skills, providing them disease specific screening and management protocols and giving NPHWs the ability to prescribe from a restricted list of medications, in consultation with physicians, where available. One of the bottlenecks in task-shifting is the inability of NPHWs to prescribe or titrate dosage of medication in many LMICs. Countries in South Asia do not allow NPHWs to prescribe medication. The ability to prescribe a restricted list of medications enables NPHWs to provide patient centred care at the community level [44]. A trial done in rural India showed that NPHWs could successfully prescribe and administer antibiotics for neonatal infections and the intervention reduced neonatal sepsis related mortality from 16.6% to 2.8% [14]. Health systems will also need to be restructured to cater to the needs of NCDs with provision of equipment such as sphygmomanometers, glucometers and weighing scales; evidence based protocols and algorithms which are easy to follow, cost-effective drug distribution systems with adequate supply of drugs; and basic population health surveillance systems. Treatment schedules should be simple to administer and not require extensive titration [45].

The World Health Organisation, in consultation with a wide range of experts has formulated a set of 22 recommendations that provide guidance to the task-shifting approach [46]. While these guidelines were developed in the context of the HIV AIDS epidemic in Sub Saharan Africa, the recommendations have implications for a range of health services including management of NCDs. The concept of task-shifting is not easily acceptable by all healthcare professionals [47]. Some view task-shifting as a competition between various healthcare providers [48] and some view it as being unsafe for patients without close physician supervision.[49] The 60th General Assembly of the World Medical Association (WMA) in 2009 passed a resolution on task-shifting, stating that while it is a short-term solution to physician shortages in LMIC, it should occur in close consultation of physicians with patient safety as a central goal. The WMA recommends further research on models of care with a physician coordinated ‘task-sharing’ approach, rather than a task-shifting model of care [50], [51].

All the studies reviewed focussed on a single risk factor or disease and no study applied the task-shifting model to a broad range of conditions, even those with similar risk factors. Given the lack of physicians in LMIC, especially in rural and remote regions, it would be worthwhile for Policy Makers to consider training and providing protocols to NPHWs to screen individuals at the community level and refer them to physicians when appropriate. A flagship project integrating NCD management has recently been piloted by the Government of India where NPHWs screen individuals for common NCD risk factors such as tobacco use, physical activity, blood sugar, blood pressure, weight, height and body mass index and refer patients at high risk to physicians at the local health centre. This project, which is yet to be evaluated, would serve as a model for integrated disease management using a multi-disciplinary team of health care providers including physicians and NPHWs.

Operational research is needed to understand issues relating to quality of care provided, patient satisfaction and importantly, given likely concerns over safety and effectiveness, health outcomes. Given that NPHWs are seen as a potentially low-cost and sustainable option for the management of NCDs in resource constrained settings, future studies should also routinely incorporate cost-effectiveness analyses. None of the studies reported process evaluation data, a critically important component to understand contextual factors associated with uptake of the intervention than may affect scale-up potential. The role of incentives and remuneration was not discussed in the studies reviewed and further research on the optimal workforce conditions is needed. A factor likely to significantly influence the feasibility of these initiatives is acceptability to patients and communities. By expanding the role given to health workers in managing chronic illness we need to better understand for instance how patients might balance potential concerns over safety and efficacy with factors such as lower costs and improved availability and access, as well as culturally appropriate care. This review was restricted to peer-reviewed articles and to the English language; hence we may have missed studies published in the grey literature and those published in languages other than English. We did not identify any study which reported negative results suggesting the possibility of significant publication bias. The majority of study designs reviewed were of inadequate quality, hence future research methods should include robust evaluations of such strategies.

Our review indicates that task-shifting is a viable and successful model and is potentially cost-effective and clinically effective for the management of NCDs. For a task-shifting model of care to function optimally several changes need to be made at the health policy and health systems level including scaling up training programs for NPHWs, provision of standardized protocols, adequate equipment and drug supply, integration of NPHWs as part of a multi-disciplinary team with support from physicians, and consultation with regulatory bodies such as the medical and nursing councils. With such systems supports in place there are substantial opportunities for major improvements in healthcare quality and outcomes for NCD management in LMICs.

## Supporting Information

Protocol S1 **Protocol for the systematic review.**
(DOCX)

Checklist S1 **PRISMA Checklist.**
(DOC)
