# Supplementary material for: Task Shifting for Non-Communicable Disease Management in Low and Middle Income Countries – A Systematic Review
Source: PLoS One. 2014 Aug 14;9(8):e103754. doi: 10.1371/journal.pone.0103754 (PMC4133198; doi:10.1371/journal.pone.0103754)
Supplement: Protocol S1 — Protocol for the systematic review. (DOCX) [file pone.0103754.s001.docx]

# PROTOCOL - Task shifting for non-communicable disease management in low and middle income countries – a systematic review

**Background:**

**The burden of NCD in low and middle income countries**

Non communicable diseases (NCDs) are the leading cause of death and disability worldwide. NCDs already disproportionately affect low and middle income countries (LMIC) where nearly 80% of these deaths (29 million) occur. NCD has not only become the leading cause of disease burden, but also occurs at a much younger age, thereby contributing disproportionately to lost potential years of healthy life, as well as lost economic productivity[[1](#_ENREF_1)]. Over the coming decades, the prevalence of NCD is expected to increase as the population ages[[2](#_ENREF_2)]. Over the next 15 years, it is anticipated that global cost of CVD will be US$47 trillion, this includes direct healthcare costs and productivity loss from disability or premature death, or time loss from work because of illness or the need to seek care[[3](#_ENREF_3)].

**The role of health services to prevent and manage NCDs**

In most countries, primary care physicians are first point of contact and the main providers of healthcare for individuals with NCDs. In LMIC too few doctors exist and physician workforce disparities for rural and remote regions are very substantial[[4](#_ENREF_4), [5](#_ENREF_5)]. In this context, there is an urgent need for new models to deliver health care. There is a need to develop a workforce that is structured around the community and consumer needs.

**The potential for task shifting in regions with a critical shortage of physicians**

In response to the health workforce shortage, appropriate strategies for task shifting from doctors to trained non-doctor agents have been developed and implemented to alleviate the chronic shortages specifically in (but not limited to) response to HIV/AIDS[[6](#_ENREF_6)]. *Task Shifting* describes a situation where a task normally performed by a physician is transferred to a health professional with a different or lower level of education and training, or to a person specifically trained to perform a limited task only, without having a formal health education[[7](#_ENREF_7)]. Task shifting may be facilitated by medical technology, which standardizes the performance and interpretation of certain tasks, therefore allowing them to be performed by non-physicians or technical assistants instead of physicians. This has typically been done in close collaboration with the medical profession[[8](#_ENREF_8)]. Task-shifting can result in substantial cost and physician time savings without compromising the quality of care or health outcomes for patients[[9-12](#_ENREF_9)].

The earliest documented study involving task-shifting occurred in The Democratic Republic of Congo in 1970s-80s where auxiliary nurses took on the role of providing healthcare due to a shortage of trained health workers. Other low and middle income countries in Africa and South Asia have recently started trialing this new model of care with NPHWs [[11](#_ENREF_11), [13](#_ENREF_13), [14](#_ENREF_14)] and nurse practitioners[[15](#_ENREF_15)]. There are very few studies that have focussed on the role of NPHW in managing NCDs, the few studies conducted till date have focussed on single risk factor or disease management[[7](#_ENREF_7), [15](#_ENREF_15)] rather than integrated disease management. There is limited evidence to show the influence of task shifting on the quality of care, acceptability by patients, costs involved and the impact of implementations.

**Review questions**

The aim of this paper is to conduct a systematic review of studies looking at ‘Task shifting’ for the management of NCD in low and middle income countries. The specific review questions are:

#### Does task-shifting improve health care effectiveness?

#### Does task-shifting improve cost-effectiveness?

#### What are the barriers and enablers that influence the effectiveness of task-shifting initiatives?

**Methods**

**Inclusion criteria**

Population: Non physician health care workers (NPHWs), community care givers, nurses working in low and middle income countries for NCD management

Intervention: task-shifting defined as ‘allocation of NCD management of patients to the least costly healthcare worker capable of doing the task’. Studies where a task normally performed by physicians is shifted to a different cadre of health care provider. Disease conditions will be limited to non-communicable disease: cardiovascular disease, diabetes mellitus, hypertension, cancer, chronic obstructive pulmonary disease, respiratory mental health

Comparison: usual care

Outcome: Change in treatment or health of participants, improved healthcare, improved knowledge of NPHWs

Types of studies: The review will include intervention studies using the following methods: RCTs/ before after studies and quasi-experimental studies

Exclusion criteria: Studies involving health education or health promotions and hospital based studies will be excluded

**Search Strategy**

A search strategy with the following terms will be used “Task-shifting”, “Non-physician healthcare workers”, “Community healthcare worker”, ”Hypertension”, “Diabetes”, “Cardiovascular disease”, “mental health”, “depression”, “chronic obstructive pulmonary disease”, “respiratory disease”, “cancer”. The following databases will be reviewed: Medline via PubMed and the Cochrane library

**Data collection**

Two authors will review the literature and extracted the data independently. In case of a disagreement about the inclusion of a paper, a third author will be asked to adjudicate. The references of all the included papers will be checked for additional relevant papers. If a study is reported in two journals, the article with the maximum detail will be chosen. If needed, details will be gathered from more than one article.

**References**

1. Joshi R, et al., *Global inequalities in access to cardiovascular healthcare: our greatest challenge.* Journal of the American College of Cardiology, 2008. **52**: p. 1817-1825.

2. Australian Institute of Health and Welfare, *Cardiovascular disease: Australian facts 2011. Cardiovascular disease series.* 2011, AIHW: Canberra.

3. Bloom DE, et al., *The Global Economic Burden of Non-communicable Diseases*. 2011, HARVARD School of Public Health.

4. World Health Organization, *World Health Report 2006: Working together for health* 2006, WHO: Geneva.

5. Ministry of Health and Family Welfare, *Rural Health Statistics Bulletin, March 2010*. 2010, Government of India: New Delhi.

6. World Health Organization, *Task shifting to tackle health worker shortages*. 2007.

7. Lekoubou A, et al., *Hypertension, Diabetes Melitus and task shifting and their management in Sub-saharan Africa.* Int. J. Environ. Res. Public Health, 2010. **7**: p. 353-363.

8. 60th WMA General Assembly, *WMA Resolution on Task Shifting from the Medical Profession*. 2009, World Medical Association,: New Delhi.

9. Mdege ND, C.S., Shehzad A,, *The effectiveness and cost implications of task-shifting in the delivery of antiretroviral therapy to HIV-infected patients: a systematic review.* Health Policy and Planning, 2012. **doi: 10.1093/heapol/czs058**.

10. Buttorff C, et al., *Economic evaluation of a task-shifting intervention for common mental disorders in India.* Bull World Health Organ, 2012. **90**: p. 813-821.

11. Abegunde DO, et al., *Can non-physician health-care workers assess and manage cardiovascular risk in primary care?* Bull World Health Organ, 2007. **85**(6): p. 432-440.

12. Callaghan M, Ford N, and Schneider H, *A systematic review of task- shifting for HIV treatment and care in Africa.* Human Resources for Health, 2010. **8**(8).

13. Joshi R, et al., *The Rural Andhra Pradesh Cardiovascular Prevention Study.* JACC, 2012. **59**(13): p. 1188-96.

14. Bang A, et al., *Effect of home-based neonatal care and management of sepsis on neonatal mortality: field trial in rural India.* Lancet, 1999. **354**: p. 1955-61.

15. Labhardt ND, et al., *Task shifting to non-physician clinicians for integrated management of hypertension and diabetes in rural Cameroon: a programme assessment at two years.* BMC Health service research, 2010. **10**(339): p. <http://www.biomedcentral.com/1472-6963/10/339>.
